# Supplementary material for: Identification of photoperiod-induced specific miRNAs in the adrenal glands of Sunite sheep (Ovis aries)
Source: Front Vet Sci. 2022 Jul 22;9:888207. doi: 10.3389/fvets.2022.888207 (PMC9354845; doi:10.3389/fvets.2022.888207)
Supplement: Supplementary file 1 [file Data_Sheet_1.ZIP › Supplementary file/miRDeep2 file.zip!/miRDeep2 file/Know_Structure/SP42A1/oar-mir-134.pdf]

miRBase precursor : oar-mir-134
Total read count : 46
oar-miR-134-5p read count : 41
oar-miR-134-3p read count : 5
remaining reads : 0

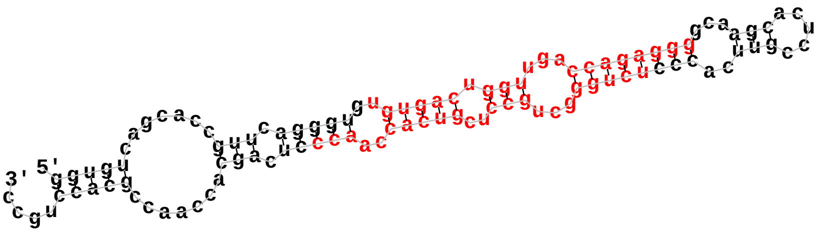

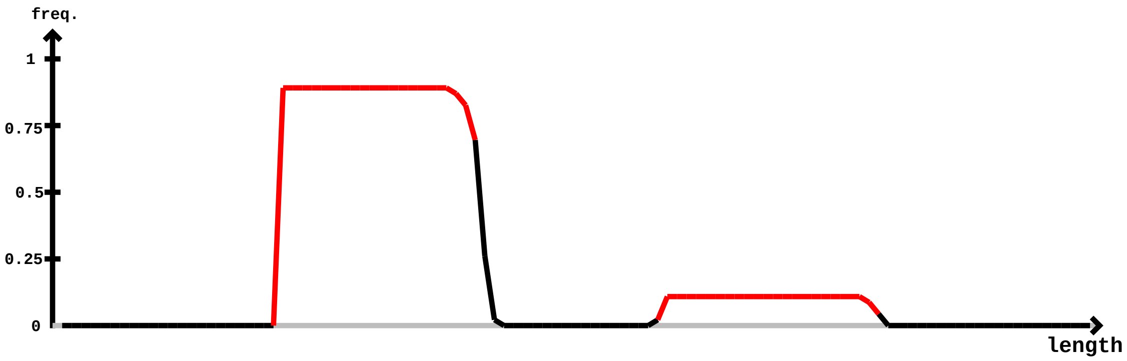


```
                                                                 oar-miR-134-3p
                      oar-miR-134-5p
5'- ggugucagcaccguucagggugugugacugguugaccagagggdcaagcacuccguucacccucugggcugccucgucaccaacccucagcaccaaccgcaccugcc   -3'    exp
    (((((.......(((.(((((..(((((.(((...((((((((...(((.....)))..))))))))...)))..)))))..))))).))).......)))))....   reads      mm         sample
    ......................ugugacugguugaccaga...................................................................   1          0          seq
    ......................ugugacugguugaccagag..................................................................   2          0          seq
    ......................ugugacugguugaccagagg.................................................................   6          0          seq
    ......................ugugacugguugaccagaggg................................................................   20         0          seq
    ......................ugugacugguugaccagagggg...............................................................   11         0          seq
    ......................ugugacugguugaccagaggggc..............................................................   1          0          seq
    .............................................................cucugggcugccucgucaccaa........................   1          0          seq
    ..............................................................ucugggcugccucgucaccaac.......................   2          0          seq
    ..............................................................ucugggcugccucgucaccaacc......................   2          0          seq
```
